# What bacterial cell death teaches us about life

**DOI:** 10.1371/journal.ppat.1010879

**Published:** 2022-10-27

**Authors:** Alex G. Johnson, Philip J. Kranzusch

**Affiliations:** 1 Department of Microbiology, Harvard Medical School, Boston, Massachusetts, United States of America; 2 Department of Cancer Immunology and Virology, Dana-Farber Cancer Institute, Boston, Massachusetts, United States of America; 3 Parker Institute for Cancer Immunotherapy, Dana-Farber Cancer Institute, Boston, Massachusetts, United States of America; Duke University School of Medicine, UNITED STATES

## Why would bacteria choose to die?

A defining feature of multicellular life is the utilization of programmed cell death (PCD) [1]. By sacrificing nonessential cells, the PCD machinery of multicellular eukaryotes defends against pathogens and cancer, while carrying out instructions for proper organismal development [2]. Once used synonymously with the term apoptosis, PCD processes in animals are now divided into separate pathways of apoptosis, necroptosis, pyroptosis, and others [3,4]. The manner in which a cell dies reflects the external inputs and underlying signal transduction processes of a cell, ultimately influencing how its death is sensed by the organism as a whole [4]. Despite major advancements in the field over the last half century, many questions remain about the evolution and functions of PCD [3].

Bacteria are commonly perceived of as unicellular organisms, so at first glance, the use of PCD seems paradoxical [5,6]. However, it is now widely accepted that bacteria and other single-celled eukaryotes commit forms of cell suicide as adaptive processes within the multicellular community that they live [5–8]. A major driving force for the evolution of bacterial PCD is warfare waged against viral invaders (bacteriophages or phages) [9–12]. Through a process called abortive infection (Abi), bacteria inhibit essential cellular processes to induce growth arrest and protect the population of neighboring cells [12]. These processes are thought to either assist with other immune mechanisms (restriction–modification and CRISPR-Cas systems) by inducing cellular dormancy, or through cellular self-destruction provide a last-ditch effort to reduce phage replication [10]. Often this mechanistic division is poorly defined, but like the PCD of multicellular eukaryotes, bacterial Abi employs host-directed mechanisms to fend off pathogens. Thus, bacterial Abi provides a window to further understand evolution of functionally analogous host-targeting systems in eukaryotes.

Here, we describe mechanisms of cell suicide shared between humans and bacteria. We focus on pyroptosis—a type of inflammatory cell death used for pathogen defense in mammals [4]. Pyroptosis is executed by gasdermin pore-forming proteins and typically results from activation of the inflammasome, a supramolecular complex that senses pathogen infection from within cells [13]. Using the recent discoveries of gasdermins in bacterial and fungal species as a case study, we highlight how an evolutionary perspective can yield insights into the mechanisms of human cell death [14–16].

## Where does the cell death machinery come from?

Shortly after initial draft sequencing of the human genome, exciting explanations for the evolution of cell death machinery began to emerge [17,18]. Across evolution, vertebrate genomes experienced a growth in the complexity of apoptosis-associated proteins or protein domains. This trend was apparent for putative “adaptors” including the Toll/interleukin-1/resistance (TIR) gene and the death domain (DD) proteins, NACHT family NTPase pathogen sensors, cysteine-aspartic proteases (caspases) critical for apoptosis and pyroptosis, and others. Homologs of several of these domains including TIRs, NACHTs, and caspases were also observed as present in bacterial genomes. These bacterial proteins were sometimes fused, indicating connectivity in ancient signaling pathways, but whether these pathways bore functionally analogy to mammalian cell death pathways was unclear [17,18].

Recent rapid growth in the number of microbial genome sequences and functional studies have illuminated the origins of cell death machinery and their role in bacteria. TIR domains, once thought of as solely adaptors, are now known to possess enzymatic function in bacteria where they participate in phage defense [19]. The DD adaptor domain, once thought to be absent from bacteria, has now been described in many bacterial lineages with predicted roles in biological conflict [20]. NACHT domains, a major feature of inflammasome sensors in mammals, are widespread in bacteria where they couple to diverse effectors to defend against phages [21–23]. And, lastly, gasdermin proteins, only recently tied to cell death processes in mammals, are present in bacterial genomes where they are activated by caspase-like proteases and defend against phages [14]. These observations indicate that mammalian cell death machinery is not only ancient but also participates in functionally analogous processes in bacteria.

## The first cut is the deepest cut?

**Fig 1:**
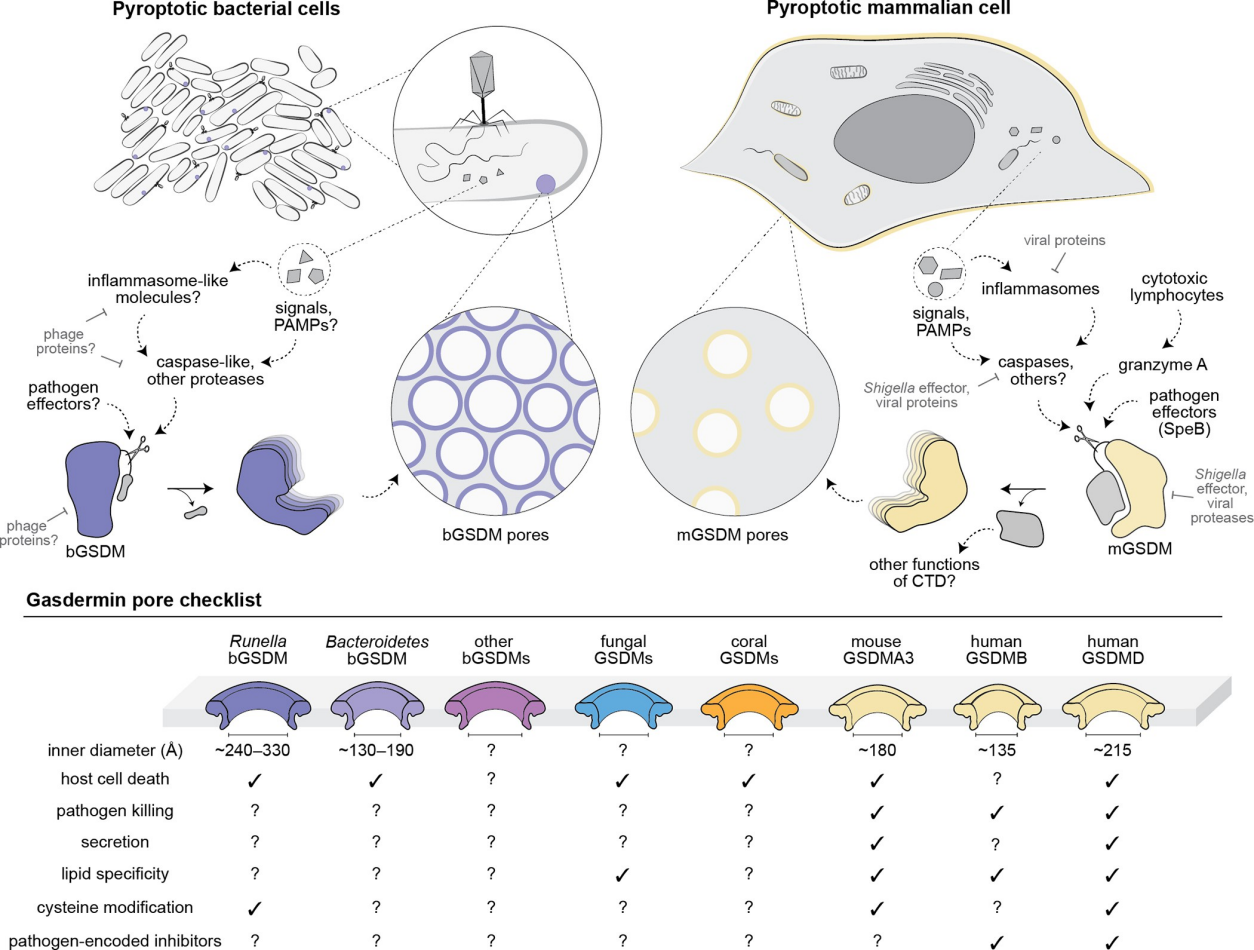
Pyroptosis across the tree of life. The top model depicts pyroptotic bacterial and mammalian cells with shared and unique signaling pathways. In this case, bacterial pyroptosis is induced by phage infection and unknown PAMPs or other signals that trigger bGSDM activation. Mammalian pyroptosis is induced by intracellular bacteria (and viral infection; not shown), leading to mGSDM activation through PAMPs or other signals, and pore formation on the plasma membrane, bacterial membranes, and mitochondria. Examples of known and predicted pathogen-encoded inhibitors are indicated with flat arrowheads. The bottom checklist depicts relative sizes of gasdermin pores across the tree of life and select features. The bacterial species are abbreviated to *Runella* (*Runella zeae* IMG ID 2525253496) and *Bacteroidetes* (unclassified *Bacteroidetes* metagenomic isolate IMG ID 2806880301). bGSDM, bacterial gasdermin; CTD, C-terminal domain; mGSDM, mammalian gasdermin; PAMP, pathogen-associated molecular pattern.

Pyroptosis was named from the Greek *pyro* meaning fire and *ptosis* meaning falling. Later discovery of the gasdermin proteins as crucial executioners of the pathway led to redefinition of pyroptosis as gasdermin-mediated cell death [13]. There are six gasdermins encoded in the human genome, and all but one of them are comprised of two roughly equally sized domains, wherein proteolytic cleavage between the domains (typically by a caspase) causes activation. The outlier, pejvakin, a deafness-associated gene, has a shorter C-terminal domain (CTD), and it remains unclear whether this protein is processed or forms pores [13]. Cleavage of any other gasdermin triggers pore formation through liberation of the autoinhibitory CTD and oligomerization of the lipophilic N-terminal domain (NTD) within membranes (**Fig 1**) [13]. Cleavage specificity was traditionally thought to be dictated by a tetrapeptide motif (XXXD) within the linker, occurring after the final aspartic acid residue. However, recent structural studies have revealed the mechanism to be more complicated, wherein at least certain gasdermins are recognized by an “exosite” within the CTD [24,25].

Bioinformatic identification of gasdermins in bacterial and fungal genomes yielded the surprising finding that these proteins are much shorter than mammalian counterparts and that homology is restricted to the lipophilic NTD, in a manner similar to pejvakin [14,15]. This suggested that bacterial gasdermins (bGSDMs) may be autoactive or might have a divergent mechanism of autoinhibition. The first possibility was quickly ruled out by expression of the full-length bGSDMs in *E*. *coli* where they were not toxic to cells. X-ray crystal structures of multiple bGSDMs unambiguously proved the latter case to be true. In the structures, a short, approximately 20–amino acid peptide wraps around the core of the bGSDM, restraining it in an inactive conformation and making inhibitory contacts like those observed between the mammalian NTD and CTD [14]. Yet, unlike the mammalian CTD, this peptide achieves its role with about 10× fewer amino acids than the mammalian CTDs. This observation motivates further analysis of pejvakin and raises the questions of why did the human CTD grow so much in size over evolution and what is accomplished by this extra mass? Part of this might relate to exosite recognition by activating proteases, but other possibilities could exist. For example, in the complement pathway of mammalian immunity, protease cleavage of the protein C3 yields two functional units: the C3a anaphylatoxin and the C3b opsonin [26]. It remains to be tested whether there are other important functions of the gasdermin CTD.

A hallmark of bacterial genomes is a tendency to cluster functionally related genes within linear genetic operons. As a result, while mammalian caspases and the target protein substrates typically exist in isolated chromosomal loci, bacterial gasdermins are often encoded immediately adjacent to caspase-like and other proteases within genomic neighborhoods [14]. Building on this finding, in vitro reconstitution of gasdermin cleavage confirmed a direct relationship between gasdermin–protease pairs. While all caspases studied to date cleave after an aspartic acid residue (or after arginine or lysine in distantly related relatives; [27]), bGSDMs are cleaved after leucine or arginine residues approximately 20 amino acids from the C-terminus [14]. This is the first example of a caspase-like protein cleaving after a hydrophobic residue and supports the notion that caspase proteins share a common protein fold rather than substrate specificity [27]. Mutational analysis of amino acids flanking each side of the cleavage site of a bGSDM indicated that only the leucine and adjacent glycine residues were essential for cleavage targeting [14]. How then does the caspase-like protease recognize the gasdermin? To investigate this further, chimeric bGSDMs were prepared with the eight amino acids surrounding the functional cleavage site inserted into predicted cleavage loops of other bGSDMs. These chimeras were not cleaved by the targeted caspase-like protease, suggesting that bGSDM recognition by proteases might also involve an exosite like in mammals [24,25]. However, without a large CTD like the mammalian gasdermin, the bGSDM exosite is likely within the NTD. Taken together, these findings imply that gasdermins and caspase-like proteases have a deeply conserved relationship in PCD across the tree of life.

## Membrane pores: The oldest trick in the book?

Bacteriophages are the most abundant biological entity on the planet, with lytic phages resulting in endless cycles of bacterial cell death [11]. Phage lysis is accomplished canonically by at minimum two proteins: a holin and an endolysin, which act in tandem to form a “hole” in the inner membrane and then degrade the cell wall, respectively [28]. Given the abundance of phages, membrane rupture through “holes” or pores may be the most common demise of cells on the planet [29]. Bacteria have, of course, evolved other ways to control cell death; in addition to employing pore-forming proteins, bacterial Abi employs an arsenal of weapons that target every type of cellular macromolecule and certain metabolites, thereby shutting down essential cellular processes [12].

In the realm of mammalian PCD, membrane pores are also of special significance [30]. In apoptosis, mitochondria outer membrane permeabilization (MOMP) is executed by effectors of the BCL-2 family, Bax and Bak, which form toroidal pores and induce release of cytochrome C, leading to downstream signaling events [30]. In necroptosis, activation and oligomerization of the pseudokinase MLKL at the plasma membrane results in membrane permeabilization and cell death [30]. Yet, out of all the cell death processes, the gasdermins are the most emblematic pore formers and have so far received the most attention by structural methods. Electron microscopy has demonstrated that activated gasdermin protomers oligomerize into large (135 to 215 Å inner diameter) membrane pores to induce pyroptotic cell death [31]. These pores induce plasma membrane rupture and in certain cases are customized for secretion of signaling molecules [32]. By virtue of their propensity to bind the cardiolipin lipid that is present in bacteria and mitochondria (“the bacteria of our cells”), gasdermin pores may also permeabilize intracellular bacteria and mitochondria for direct pathogen killing or MOMP, respectively.

Reconstitution of bGSDM pores yielded several insights into the protein family [14]. First, unlike the mammalian gasdermins studied so far, active bGSDMs bind to and permeabilize membranes lacking cardiolipin or phosphoinositides. This result is surprising since cardiolipin is present in bacterial host membranes and could suggest that lipid-specific interactions are an evolutionary innovation of mammalian and fungal gasdermins [15]. Second, the majority of bGSDMs are covalently modified by a palmitoyl thioester at an N-terminal cysteine residue, and when present, removal of this lipid modification reduces the efficiency of pore formation. Drugs and metabolites that react with cysteine residues of human gasdermins are known to inhibit their processing and pore formation, raising the question of how these lipid modifications aid in pore formation and whether they might be evolutionarily conserved [13,33]. Finally, the two bacterial gasdermins studied so far assemble into pores with inner diameters of 130 to 190 Å and 240 to 330 Å and tend to form supramolecular mesh-like assemblies in vitro (**Fig 1**). The fact that bacterial gasdermins pores can be >100 Å wider than human gasdermin counterparts may reflect tailoring for specific functions in secretion or cell death that await further exploration.

**Fig 2:**
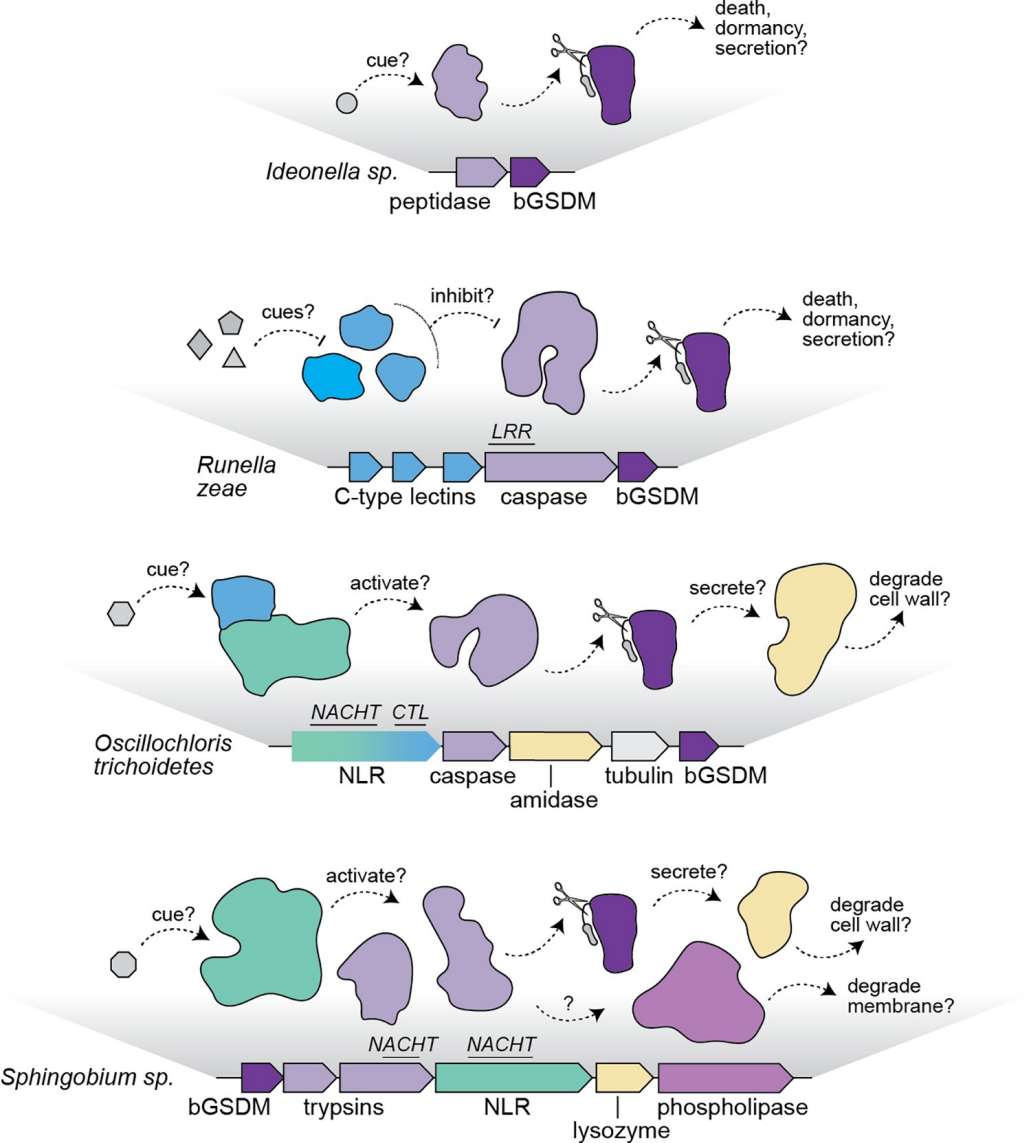
bGSDM operons suggest a diversity of unexplored signaling mechanisms. From top to bottom, operons are from *Ideonella* sp. (IMG ID 2684147428), *Runella zeae* (IMG ID 2525253496), *Oscillochloris trichodetes* (IMG ID 650112614), and *Sphingobium* sp. (IMG ID 2855415098). The *Sphingobium* operon is of a similar architecture to the *Lysobacter enzymogenes* operon, which defends against phages when expressed in *E*. *coli*, but lacks the closely associated genes encoding lysozyme and phospholipase proteins. Genes are illustrated with potential roles in the upstream activation of bGSDM signaling or as downstream effectors. Select domains with potential roles in pathogen sensing are italicized above genes. Genes and domains are abbreviated as follows: peptidase (Peptidase U49), caspase (caspase-like including Peptidase C14 and CHAT), C-type lectins or CTL (typically annotated as formylglycine-generating enzymes or FGS), LRR (leucine-rich repeats), NLR (nucleotide-binding oligomerization domain (NOD)-like receptor), NACHT (NAIP, CIIA, HET-E, and TP1 domain), amidase (N-acetylmuramoyl-L-alanine amidase), tubulin (FtsZ), and trypsin (trypsin-like).

Though no clear homologs of Bax/Bak and MLKL proteins have been identified in bacterial genomes, there is still much to learn through a comparative evolutionary analysis. For instance, MLKL-like proteins are present in fungi where they control PCD events related to heterokaryon incompatibility [34]. These MLKL-like domains are sometimes fused to proteases associated with fungal gasdermins, suggesting overlap between necroptotic and pyroptotic cell death processes [14,16]. The targeting of mitochondrial membranes in apoptosis may also be a remnant of the evolutionary events during the endosymbiosis of a bacteria that became our mitochondria [6]. In a remarkable example, researchers demonstrated that active Bax/Bak can substitute for the holin gene in a phage genome and enable lytic activity [35]. Analysis of the bacterial gasdermin genomic operons reveals further functional analogies to the phage holins, wherein endolysin-like proteins (lysozymes, amidases) are sometimes encoded nearby (**Fig 2**). Given the role of bacterial gasdermins in phage defense, this observation raises the possibility that holin–endolysin mimicry is a strategy by which bacteria alter the clock of the phage life cycle to abort infection. Utilization of membrane pores in bacteria–phage conflict may have provided a template for the evolution of multiple cell death processes in eukaryotes.

## What activates bacterial gasdermins and do they always cause cell death?

A major open question regarding gasdermin signaling relates to identification of the molecular cues responsible for pathway activation. In one of the best-studied examples, the mammalian NAIP-NLRC4 inflammasome is activated by direct binding to bacterial-derived ligands such as flagellin, leading to downstream gasdermin activation [36]. However, multiple activating ligands have been proposed for other inflammasomes, and sometimes ligands bypass the NOD-like receptor (NLR) inflammasomes for “noncanonical” caspase activation [36]. Since bGSDMs occur in distinct operon organizations, a diversity of activation mechanisms is also anticipated in bacteria (**Fig 2**). bGSDMs are closely linked to protease-encoding genes, and these systems fall into two categories: those for which no discernible cell death occurs during coexpression (the majority) or those whose coexpression leads to cell death. The latter “autoactive” bGSDMs were critical for in vitro reconstitution experiments, but such constitutive cell death is unlikely to represent normal physiology. These systems might either be activated by a molecule present in *E*. *coli* and not their native host, or they are blocked by another host protein that restrains protease activity. The latter possibility might be raised for CTLs, which cluster nearby some bGSDM-encoding genes and may be immune receptors (**Fig 2**) [20,37,38].

In the most complex cases, bGSDMs are just one component of multigene systems that encode domains present within inflammasome sensors (**Fig 2**). Preliminary analysis of one such system from *Lysobacter enzymogenes* defined a role for these operons in phage defense, though the activating cue from phage infection remains unknown [14]. These inflammasome-like operons come in several forms and are sometimes linked to other genes whose products encode potential cell wall degrading effectors. Such systems may be analogous to phage holin–endolysin systems, but these effectors also bear some similarities to type VI secretions systems involved in bacteria–bacteria conflict [39]. This suggests that bGSDMs may not always be the final effectors of cell death signaling, as with the case of human Ninj1 [40]. It is worth asking whether bGSDMs participate in other biological conflict systems besides phage defense and whether their activation always results in cell suicide. Indeed, human gasdermins do not always induce cell death and are sometimes activated in live cells for the secretion of extracellular signaling molecules [41]. Understanding the interplay of genes within bGSDM operons will surely reveal other unknowns about human inflammasomes.

The study of bGSDMs illuminates the evolutionary history of the gasdermin protein family, revealing new insights into the mechanism of autoinhibition, cleavage, and pore formation. The sophisticated nature of bGSDM systems and the information provided by genomic clustering of related genes has the potential to yield insights into regulation of pathogen sensing and PCD. The deployment of gasdermins in antiviral defense across the tree of life indicates that pathogen-encoded anti-gasdermins await discovery (**Fig 1**). Gasdermins are but one component of the cell death toolkit with bacterial origins, and the mammalian cell death machinery with TIRs, NACHTs, and DDs undoubtedly has many more secrets hidden in bacteria. In this way, bacterial cell death will continue to teach us many more things about life.
